# LncRNAs as epigenetic regulators of epithelial to mesenchymal transition in pancreatic cancer

**DOI:** 10.1007/s12672-022-00522-0

**Published:** 2022-07-11

**Authors:** Yan Ma, Yang Di, Qiuyue Li, Qilin Zhan, Xiaomeng He, Shanshan Liu, Heng Zou, Christopher Corpe, Litian Chen, Jin Wang

**Affiliations:** 1 Shanghai Public Health Clinical Center, Fudan University, 2901 Caolang Road, Jinshan District, Shanghai, 201508 People’s Republic of China; 2 Department of Pancreatic Surgery, Huashan Hospital, Fudan University, Shanghai, China; 3 King’s College London, Nutritional Science Department, 150 Stamford Street, Waterloo, London, SE19NH UK; 4 Department of Hepatobiliary Surgery, Shanghai Jiaotong University School of Medicine Xinhua Hospital, Kongjiang Road 1665, Shanghai, China

**Keywords:** Pancreatic cancer, lncRNA, Metastasis, EMT, TGF-β/Smad, HIF-1α

## Abstract

Pancreatic cancer is the leading cause of cancer-related mortality because of tumor metastasis. Activation of the epithelial-to-mesenchymal transition (EMT) pathway has been confirmed to be an important driver of pancreatic cancer progression from initiation to metastasis. Long noncoding RNAs (lncRNAs) have been reported to exert essential physiological functions in pancreatic cancer progression by regulating the EMT program. In this review, we have summarized the role of EMT-related lncRNAs in human pancreatic cancer and the potential molecular mechanisms by which lncRNAs can be vital epigenetic regulators of epithelial to mesenchymal transition. Specifically, EMT-activating transcription factors (EMT-TFs) regulate EMT via TGF-β/Smad, Wnt/β-catenin, and JAK/STAT pathways. In addition, the interaction between lncRNAs and HIF-1α and m6A RNA methylation also have an impact on tumor metastasis and EMT in pancreatic cancer. This review will provide insights into lncRNAs as promising biomarkers for tumor metastasis and potential therapeutic strategies for pancreatic cancer.

## Introduction

Pancreatic cancer is the third-leading cause of cancer-related mortality in the USA. It also has a high mortality rate, which has been increasing in incidence in recent years due to the lack of effective tools for early diagnosis, pancreatic tumor metastasis, poor response to existing conventional strategies and drug resistance [1–4]. Although advances in screening, prevention, diagnosis and treatment over the past years have diminished cancer incidence and mortality rates and have improved the quality of life of patients, the 5 year survival rate at the time of diagnosis is still only 10% in the USA [5] because approximately 80–85% of patients present with either unresectable pancreatic tumors or tumor metastasis [6]. Tumor metastasis is a complex multistep process that includes epithelial-to-mesenchymal transition (EMT), migration via blood vessels or lymphatic vessels to escape the primary site and then invasion into the surrounding or distant tissues or organs [7, 8]. Although the histomorphology and genetics of pancreatic carcinogenesis have been well elucidated [9], the molecular mechanisms that promote the metastatic spread of pancreatic cancer have not yet been clarified.

Epithelial to mesenchymal transitions are reversible biological processes involved in various physiologic and pathologic processes in which epithelial cells lose their characteristics, such as polarity and tight junction contacts. During EMT, cells also undergo cellular, molecular, and biochemical changes to gain motility, migratory potential, and invasive properties, eventually becoming mesenchymal stem cells. These biological characteristics enable tumor cells to acquire the ability of metastasis, invasion, antiapoptosis and drug resistance, which play an important role in the malignant progression of tumors and are considered a hotspot with regard to the molecular mechanism of tumors [10]. Based on their biological contexts, EMT can be classified into three distinct types. Unlike type I and type II EMT, type III EMT retains the characteristics of epithelial cells and is associated with cancer progression and metastasis, contributing to increased cancer cell invasiveness [11]. These malignant behaviors have been significantly correlated with early EMT in premalignant lesions. In the process of EMT, tumor cells have changed both in morphology and biological function and acquire invasive, metastatic and chemoresistant properties. Pancreatic ductal adenocarcinoma (PDAC), a glandular epithelial malignancy, possesses multiple developmental mechanisms that promote the occurrence of EMT. EMT is a highly coordinated process triggered by many signaling pathways that have been identified in the transformation of epithelial cells to mesenchymal cells such as TGF-β/Smad, Wnt/β-catenin, Hedgehog, and HIF signaling pathways [12–14]. Cells undergoing EMT progressively lose the expression of E-cadherin (epithelial cell surface biomarkers) in epithelial cell junctions and gain the expression of the mesenchymal marker vimentin (mesenchymal cell surface biomarkers). Multiple EMT-transcription factors (TFs) regulate the EMT process, and the most widely studied are the Snail, Twist, and ZEB families [12]. The abnormal expression of TFs can inhibit the expression of E-cadherin and promote the expression of N-cadherin and vimentin. EMT-activating transcription factors (ZEB, Snail and Twist) are also considered to be the most critical regulators during the development of EMT [11]. In recent years, emerging studies have demonstrated that transcriptional or posttranscriptional regulatory networks, such as ncRNAs (miRNA, lncRNA, and circRNA), can mediate the progression of EMT [15]. Long noncoding RNAs (lncRNAs) are common noncoding RNAs and have been reported to exert essential physiological functions in a variety of types of cancer progression to regulate the EMT program. LncRNAs are defined as transcripts of more than 200 nucleotides that can be found in the nucleus or cytoplasm and generally do not code for proteins, although some transcripts are also annotated as lncRNAs that encode small proteins [16].

## lncRNAs regulate gene expression through diverse mechanisms that mediate the transcriptional regulation of EMT

Aberrant expression levels of lncRNAs have been identified in tumor cells or tissues, such as lung cancer, colorectal cancer, gastric cancer, breast cancer, liver cancer and pancreatic cancer, and are associated with tumorigenesis, proliferation, migration, invasion, and tumor metastasis, which may exhibit tumor-suppressive or tumor-promoting functions [17]. For example, lncRNA linc00673 facilitates non-small cell lung cancer (NSCLC) proliferation, migration, invasion and epithelial mesenchymal transition by sponging miR-150-5p [18]. LncRNA TUG1 promotes tumor cell metastasis and epithelial-mesenchymal transition in a series of cancers [19–22]. LncRNAs contain a heterogeneous class of intergenic transcripts, enhancer RNAs (eRNAs), and sense or antisense transcripts that overlap with other genes. The diverse biological contributions of lncRNAs have been demonstrated, including cis- and trans-transcriptional regulation, organization of nuclear domains, regulation of mRNA processing, post‐transcriptional control and protein activity [23]. To clarify the roles of these differentially expressed lncRNAs in cancer, a more insightful description of the mechanisms governed by the key lncRNAs/miRNAs/proteins involved in EMT was reviewed.

### lncRNAs associate with gene promoters and regulatory regions and recruit protein complexes to activate or inhibit gene transcription

LncRNAs, as vital regulators of both development and disease, could play critical roles in a wide range of biological processes, including stem cell maintenance, lineage differentiation and cancer progression (cell proliferation, cell apoptosis, cell invasion, and metastasis) [17, 24, 25], and can serve as activators, inhibitors, guides, or scaffolds for their interacting proteins, DNA or RNA to regulate gene expression [23]. Although there are many molecular mechanisms responsible for EMT, it has been reported that there are two broad mechanisms through which lncRNAs exert their effect. One molecular mechanism refers to the association of lncRNAs with gene promoters and regulatory regions, where they recruit protein complexes to activate or inhibit gene transcription [26].

### lncRNAs bind to miRNAs as sponges and protect their target mRNAs from the effects of miRNAs

Together with previous reports of lncRNAs driving malignant behaviors, these observations raise the intriguing possibility that lncRNAs may seek specific pathways to promote tumor progression. For example, upregulated lncRoR could promote YAP localization to the nucleus, thus stimulating EMT‐related protein expression via the Hippo signaling pathway [27]. LncRNA HOTAIR, which has been reported in many different types of human cancers, can change the profiling of gene expression to facilitate tumorigenesis and metastasis [28, 29]. HOTAIR upregulation in gastric cancer can enhance cancer progression and metastasis by sponging miR-331-3p to regulate HER2 expression [30]. Overexpression of HOTAIR in hepatocellular carcinoma (HCC) can also promote the invasion and migration of HCC by enhancing the EMT regulatory process, in which HOTAIR upregulates ZEB gene expression by sponging miR-23b-3p [31]. Lnc01420 is upregulated in pancreatic cancer and facilitates pancreatic cancer cell proliferation, metastasis and EMT, and lnc01420 can act as a sponge of miR-494-3p to relieve the inhibition of MYC expression and promotes KRAS gene transcription through the lnc01420/miR-494-3p/MYC regulatory axis [32]. LncRNA OIP5-AS1 promotes malignancy in pancreatic cancer and silences miR-429 through the mechanism of ceRNA, leading to upregulation of FOXD1 expression in PDAC [33]. LncRNA NORAD serves as a novel competing endogenous RNA through competition for miR-125a-3p and can enhance hypoxia-induced epithelial-mesenchymal transition to promote metastasis in pancreatic cancer [34].

### LncRNAs act as a molecular scaffold to recruit their target genes to regulate gene expression through the traditional ceRNA mechanism

lncRNAs function as molecular scaffolds to recruit chromatin modifiers, thereby repressing or activating the expression of target genes, such as HOTAIR. HOTAIR overexpressed in lung cancer could recruit Polycomb Repressive Complex 2 (PRC2), a transcriptional corepressor, to repress the expression of the homeobox gene D cluster (HOXD) [28]. LncRNA GClnc1 acts as a molecular scaffold to recruit the WDR5 and KAT2A complex and modifies the transcription of target genes, consequently altering gastric cancer cell biological processes [35].

### lncRNAs modulate posttranscriptional events during gene expression such as gene alternative splicing, mRNA translation and mRNA degradation

LncRNA MALAT1 promotes premetastatic phenotypes of ovarian cancer by promoting alternative RNA splicing and differential expression of genes associated with anti-apoptosis and EMT [36]. High expression of lncRNA RP1 in breast cancer cells and tissues is closely associated with malignant progression and poor prognosis in breast cancer patients, which indicated that RP1 could promote EMT and maintain the stemness states of breast cancer cells by binding to the p-4E-BP1/eIF4E complex, reduced the translation efficiency of p27kip1 mRNA, and repressed p27kip1 protein expression [37]. LncRNAs have also become a novel research focus by regulating the degradation of mRNA. LncRNA LDLRAD4-AS1 reduces the expression of LDLRAD4 by destabilizing LDLRAD4 mRNA, which leads to upregulation of Snail and promotes EMT, thereby promoting the metastasis of colorectal cancer [38]. In conclusion, accumulated evidence suggests that lncRNAs are emerging as important transcriptional or posttranscriptional regulators.

### The interplay between m6A RNA methylation and lncRNAs in cancer

For instance, beyond the canonical pathway of lncRNA sponge miRNAs, there is another possible pathway that regulates the progression of EMT. Recent evidence has demonstrated an important interplay between m6A RNA methylation and noncoding RNA in cancer [39]. N6-methyladenosine (m6A) methylation, as one of the most common RNA modifications, has been reported to execute important functions that affect cancer progression, including proliferation, invasion and metastasis. lnc00460 promoted colorectal cancer proliferation, migration, invasion and EMT by mediating HMGA1 mRNA stability in an m6A-dependent manner [40]. The m6A modification mediated the proliferation of the cells expressing the lncRNA THOR, which enriched m6A [41]. Collectively, these results suggest that lncRNAs can regulate tumor progression through epigenetic changes. Future investigation will be required to unveil the molecular mechanisms of how lncRNAs promote tumor progression through epigenetic changes.

## LncRNAs enhance cancer progression and metastasis as vital regulators of EMT in pancreatic cancer

Several lncRNAs intimately participate in the regulation of genes involved in the EMT process in pancreatic cancer. Here, we have summarized the current understanding of the molecular mechanisms of lncRNAs mediating EMT signaling pathways, describing how lncRNAs regulate EMT and what the signal is protected from pancreatic cancer malignant development (Table 1).

**Table 1:** EMT-related lncRNAs in human pancreatic cancer

LncRNA	Expression	Metastasis	Sponge miRNA	Molecular mechanism	References
ROR	up	EMT	/	ROR/Hippo/YAP	[27]
Lnc01420	up	EMT	miR-494-3p	lnc01420/miR-494-3p/MYC/KRAS	[32]
OIP5-AS1	up	EMT	miR-429	OIP5-AS1/miR-429/FOXD1/ERK	[33]
NORAD	up	EMT	miR-125-3p	NORAD/miR-125-3p/RhoA/ROCK	[34]
MEG8	up	EMT	miR-34a/miR-203	MEG8/miR-34a/miR-203/Snail	[43]
PVT1	up	EMT	/	PVT1/P21/ZEB1/Snail	[48]
BX111	up	EMT	/	HIF-1α/BX111/YB1/ZEB1	[49]
XIST	up	EMT	miR-429	XIST/miR-429/ZEB1	[50]
DYNC2H1-4	up	EMT	miR-145	DYNC2H1-4/miR-145/ZEB1	[52]
ABHD11-AS1	up	EMT	/	ABHD11-AS1/PI3K-AKT	[58]
Lnc00462	up	EMT	miR-665	lnc00462/miR-665/TGFβR1-TGFβR2/SMAD2/3	[65]
Lnc00261	down	MET^a^	/	TGFβ-1/FOXA2/lnc00261	[69]
Lnc01133	up	EMT	/	lnc01133/AXIN2/Wnt/β-catein	[74]
HOTTIP	up	EMT	/	HOTTIP/WDR5/HOXA9/Wnt/β-catenin	[75]
DLX6-AS1	up	EMT	miR-497-5p	DLX6-AS1/miR-497 5p/FZD4/FZD6/Wnt/β-catenin	[76]
TSLNC8	up	EMT	/	TSLNC8-HuR/CTTNB1/Wnt/β-catenin	[77]
HOTAIR	up	EMT	/	HOTAIR/Wnt/β-catenin	[78]
H19	up	EMT	miR-675-3p	H19/miR-675-3p/SOCS5/STAT3	[84]
PCED1B-AS11	up	EMT	miR-411-3p	PCED1B-AS1/miR‑411‑3p/HIF‑1α	[91]
FEZF1-AS1	up	EMT	miR-142	FEZF1-AS1/miR-142/HIF-1α	[92]
ENST00000480739	down	MET^a^	/	ENST00000480739/OS-9/HIF-1α	[93]

^a^MET mesenchymal-epithelial transition

### LncRNAs are not only involved in the transcriptional regulation of EMT-TFs but also act synergistically with various transcription factors to regulate EMT in pancreatic cancer

Transcription factors play an essential role in the development of EMT, such as cell invasion, adhesion, and migration. Several lncRNAs have been reported to be involved in the regulation of EMT via the transcriptional regulation of EMT-activating transcription factors (EMT-TFs). Epithelial to mesenchymal transition is primarily initiated by a core set of EMT-TFs, including Snail (also known as Snail1), Slug (also known as Snail2), Twist-related protein 1 (Twist1), Zinc-finger E-box-binding homeobox 1 (ZEB1) and ZEB2, which can activate classic EMT and result in the disjunction of cellular adhesions, loss of epithelial cell polarity and manifestation of a mesenchymal, motile phenotype [12, 42]. For example, lncRNA MEG8, mainly induced by the TGF-β factor, is significantly upregulated in lung cancer and pancreatic cancer [43–45] and can downregulate miR-34a to repress E-cadherin expression [35]. Snail family mRNA (Snail1 and Snail2), a common EMT-TF, was specifically inhibited by miR-34a by inducing the formation of the RISC complex [46, 47], which suggested that MEG8 could indirectly activate Snail1 and Sanil2 to promote EMT progression. LncRNA PVT1 was significantly upregulated in pancreatic cancer cell lines and tissues and could promote EMT in pancreatic cancer cells by downregulating p21, which directly regulates the expression of Snail [48]. ZEB1/2, another important EMT-TF, can also directly or indirectly control the transcription of EMT-associated genes. LncRNA-BX111, induced by hypoxia, was overexpressed in pancreatic cancer and could promote the metastasis and progression of pancreatic cancer by regulating the transcriptional regulatory factor ZEB1 [49]. LncRNA XIST can indirectly enhance ZEB1 expression by sponging miR-429 and promoting cancer invasion, migration and EMT. Knockdown of lncRNA XIST counteracts the effect of ZEB1 by promoting metastasis through the upregulation of miR-429 [50], which identified the critical axis of XIST/miR-429/ZEB1 in pancreatic cancer migration, invasion and EMT. LncDYNC2H1-4 can facilitate pancreatic cancer EMT as a sponge for miR-145 to mediate the repression of ZEB1, which has been identified as a direct target of miR-145 [51, 52]. LncRNA-ROR also facilitates pancreatic cancer metastasis by acting on the ZEB1 pathway [53].

**Fig. 1:**
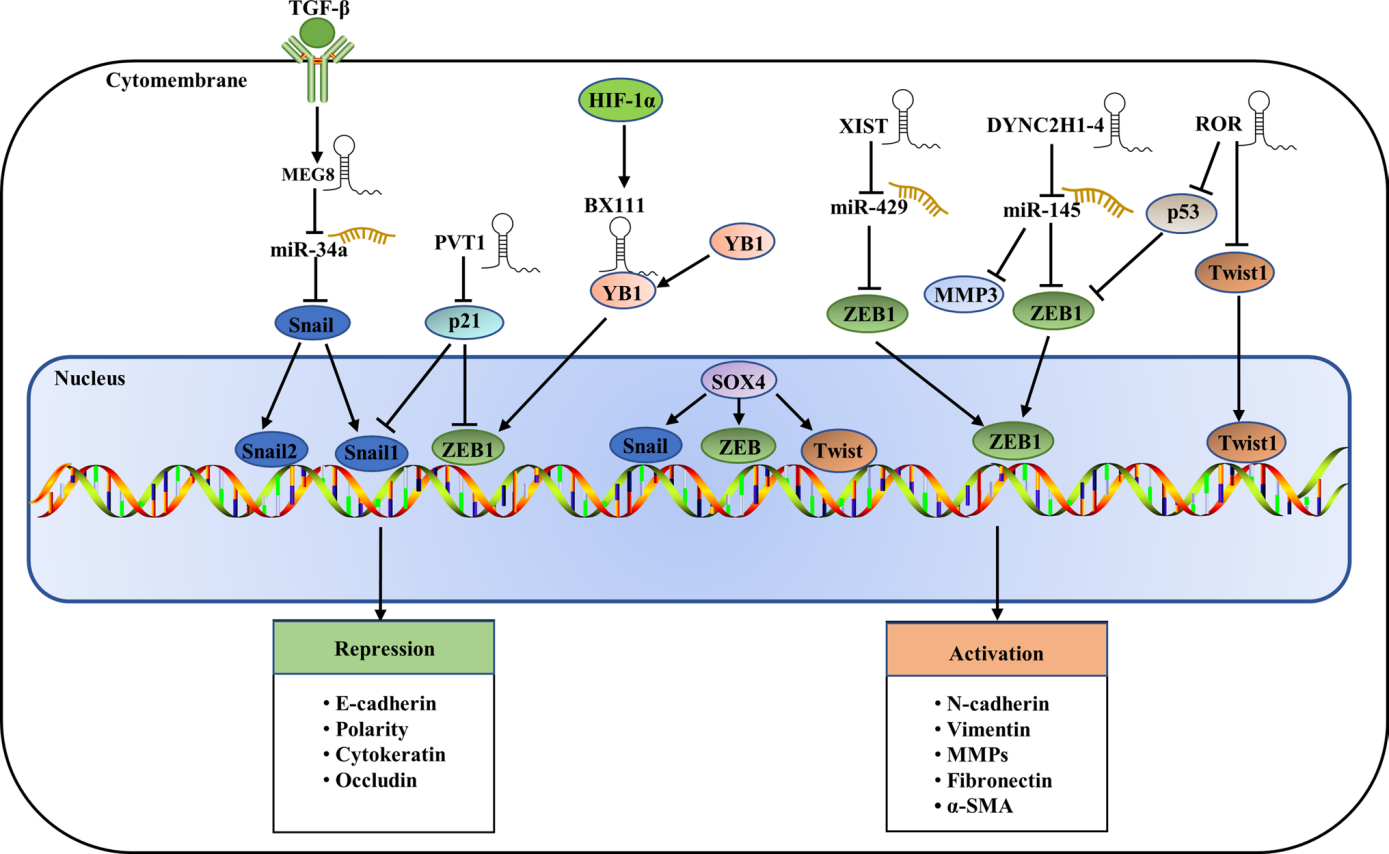
Roles and regulation of major EMT transcription factors. Epithelial mesenchymal transition is driven by Snail, ZEB and Twist transcription factors, and lncRNAs mainly act as sponges for miRNAs to regulate the expression of transcription factors

The interplay between the transcription factor SOX4 and various EMT regulators contributes to EMT [54]. SOX4 promotes the transcription of the EMT transcription factors Snail, ZEB, and Twist through intermediate protein or epigenetic modifications, resulting in enhanced expression of N-cadherin and vimentin and hence promoting EMT [55]. LncRNAs regulate EMT mainly by acting as ceRNAs for SOX4-targeted miRNAs [56] or directly targeting the promoter of SOX4 [57]. SOX4 appears to be a critical regulator of various signaling pathways and may be a crosstalk factor between signaling pathways, thereby participating in the progression of tumor EMT, such as the activation of the TGF-β/Smad2/3 and PI3K/AKT pathways [54, 58]. SOX4 promotes the transcription of the EMT transcription factors Snail, ZEB, and Twist through intermediate protein or epigenetic modifications, resulting in enhanced expression of N-cadherin and vimentin and hence promoting EMT [55]. In summary, we speculate that lncRNAs can act synergistically with various transcription factors to regulate EMT progression through multiple signaling pathways. The details of the interactions between all these transcription factors and lncRNAs are shown in Fig. 1.

### LncRNAs regulate EMT via the TGF-β/Smad, Wnt/β-catenin, and JAK/STAT pathways in pancreatic cancer

#### TGF-β/Smad pathway

**Fig. 2:**
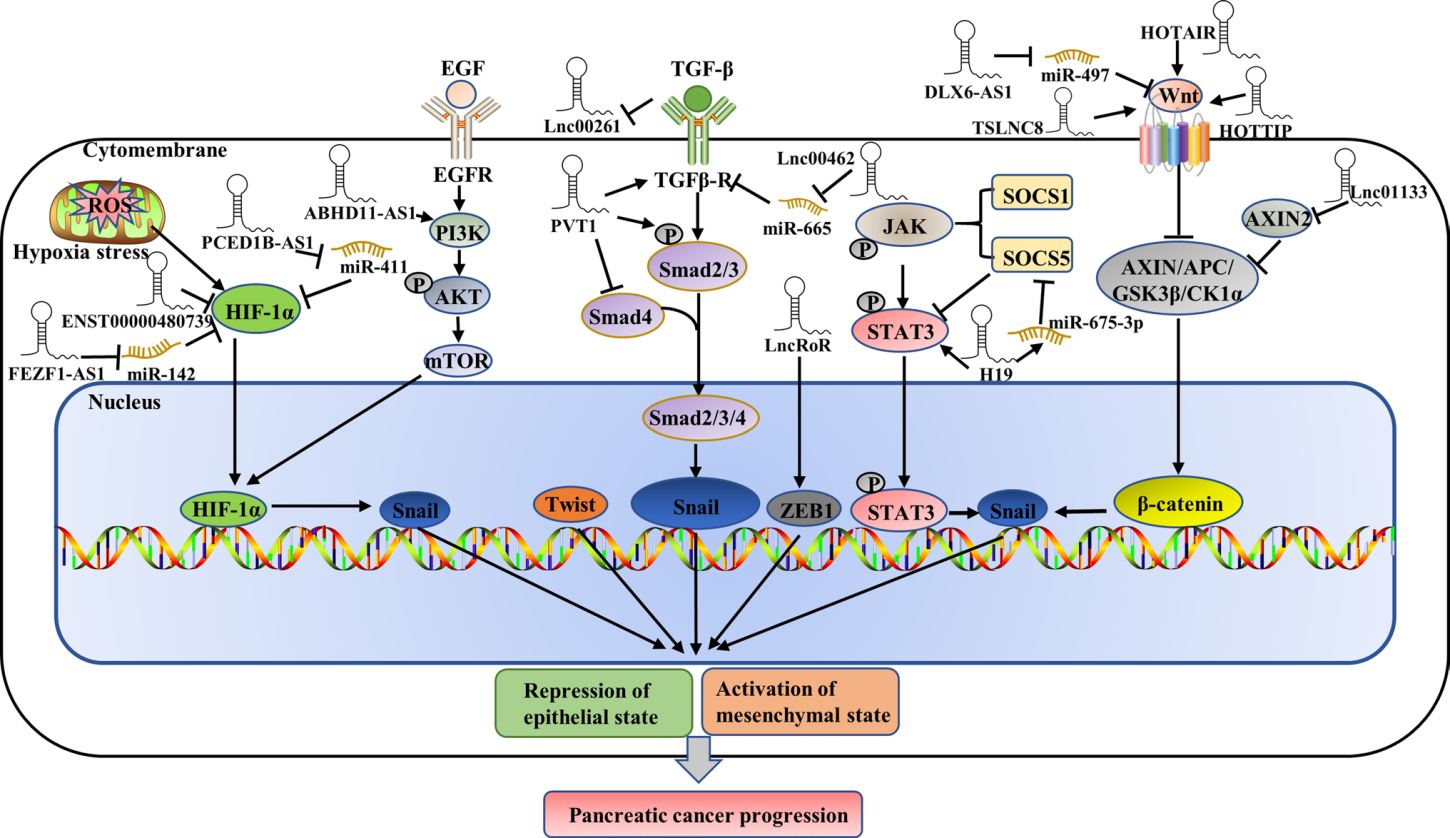
The progression of epithelial-mesenchymal transition is regulated by lncRNAs that are involved in multiple complex signaling pathways, such as the TGFβ/SMAD2/3, Wnt/β-catenin, mTOR, HIF-1α and JAK/STAT3 pathways

Epithelial to mesenchymal transition of tumors can be induced by multiple secreted cytokines from the stroma, such as transforming growth factor beta (TGF-β), hepatocyte growth factor (HGF), vascular endothelial growth factor (VEGF) and platelet-derived growth factor (PDGF) [59–62]. Among these, TGF-β is a major inducer of EMT during embryogenesis, development, cancer progression and fibrosis [63]. TGF-β can specifically bind to TGF-β type I and II receptors (TGF-βRI/TGF-βRII) on the cell surface, and TGF-β RI/TGF-β RII serine/threonine kinase activity leads to the phosphorylation of Smad2 and Smad3. Phosphorylation activates the Smad signaling cascade, leading to the nuclear translocation of Smad4, which drives the transcription of a wide range of tumor-promoting genes (Fig. 2) [64]. LncRNAs can also regulate EMT progression by activating TGF-β in pancreatic cancer. For example, lnc00462 promotes pancreatic cancer invasiveness and metastasis through the miR-665/TGF-βRI-TGF-βRII/SMAD2/3 pathway [65]. Lnc00462 promotes the progression of pancreatic cancer through the molecular mechanism of ceRNA and serves as a molecular sponge for miR-665 to upregulate TGF-βRI and TGF-βRII and activate the Smad2/3 signaling pathway to enhance the invasive ability of pancreatic cancer [65]. LncRNA XIST regulates the EGF receptor to promote TGF-β1-induced EMT in pancreatic cancer by acting as a sponge for miR-34a, which has been reported to regulate various cell processes, including EMT, in many malignant tumors [12, 66]. MEG8 can also regulate EMT progression by activating TGF-β in pancreatic cancer [35] and lung cancer [67]. LncPVT1 can augment TGF-β/Smad signaling, which sequentially induces EMT in pancreatic cancer [68]. On the other hand, lnc00261 is downregulated in pancreatic cancer and can inhibit cancer progression [69, 70], and methylation-mediated lnc00261 can participate in regulating pancreatic cancer by epigenetically inhibiting c-Myc transcription to suppress pancreatic cancer malignant progression [71]. Specifically, in response to TGF-β stimulation, lnc00261 was downregulated by TGF-β [71], which suggested that TGF-β could also regulate lncRNAs to promote EMT progression in pancreatic cancer.

#### Wnt/β-catenin pathway

The Wnt pathways can be divided into a canonical pathway (Wnt/β-catenin pathway) and a noncanonical pathway (Wnt/PCP and Wnt/Ca2 + pathways) [72]. The Wnt/β-catenin signaling pathway has been reported to be involved in the control of pancreatic cancer development [73]. In the cytoplasm, Wnt protein binds to a cell surface receptor composed of seven transmembrane curl proteins and LRP5/6 to activate the Wnt/β-catenin signaling pathway. When the Wnt/β-catenin signaling pathway is activated, it inhibits the degradation of β-catenin, which regulates the transcription of many genes, including c-Myc and cyclin D1. However, in the absence of Wnt, β-catenin, Axin, glycogen synthase kinase-3 (GSK3), adenomatous polyposis coli (APC), and casein kinase 1 (CK1) form a destruction complex in the cytoplasm, in which the complex allows β-catenin to be phosphorylated, ubiquitinated and finally degraded by the proteasome [73]. Axin and GSK3 play crucial roles in inhibiting the Wnt/β-catenin pathway. Axis inhibition protein 2 (AXIN2), which can suppress GSK3 activity and destruction complex formation and ultimately inhibit β-catenin, is involved in the regulation of cell proliferation, invasion, migration, metastasis and other functions in a variety of cancers. LncRNAs can also regulate EMT in pancreatic cancer through the Wnt/β-catenin pathway (Fig. 2). Lnc01133 derived from tumor exosomes is highly expressed in both pancreatic cancer cells and their secreted exosomes and can promote pancreatic cancer EMT via the Wnt/β-catenin pathway by silencing AXIN2 [74]. LncRNA HOTTIP was significantly upregulated in pancreatic ductal adenocarcinoma compared with chronic pancreatitis [75]. The upregulation of HOTTIP/HOXA9 enhances the Wnt/β-catenin signaling pathway, whereas silencing of HOTTIP/HOXA9 reduces the expression of the Wnt protein family (Wnt1, Wnt3a, Wnt10a, and Wnt110b) in pancreatic cancer [75]. LncDLX6-AS1, HOTAIR and lncTSLNC8 can also directly or indirectly trigger the Wnt/β-catenin signaling pathway to stimulate EMT progression [76–78].

#### JAK/STAT3 pathway

The role of the JAK/STAT3 signaling pathway in the regulation of metastasis, the transition of cancer stem cells, and chemoresistance of cancer by EMT has sparked the development of therapeutic targets [79–83]. LncRNA H19 is significantly upregulated in pancreatic cancer cell lines and facilitates pancreatic cancer metastasis and EMT by directly targeting miR-675-3p, which binds to SOCS5, a member of the SOCS protein family that negatively regulates JAK2/STAT3 signaling (Fig. 2) [84]. Additionally, abnormally expressed lncRNA PSMB8-AS1 is involved in the progression of pancreatic cancer by enhancing the STAT1/PD-L1 pathway. The upregulation of PSMB8-AS1 can promote STAT1 expression and contribute to pancreatic cancer malignant behaviors by modulating the miR-382-3p/STAT1/PD-L1 axis [85].

### The interaction between lncRNAs and HIF-1α impacts tumor metastasis and EMT in pancreatic cancer

Pancreatic cancer is a solid tumor, and hypoxia is also a common tumor microenvironment, resulting in the upregulation of HIF-1α. Hypoxia-inducible factor-1α (HIF-1α) is a master transcriptional regulator of the adaptive response to hypoxia that plays an essential role in various metabolic activities, including embryonic vascularization, tumor angiogenesis and pathophysiology of ischemic disease. LncRNA NORAD can modulate hypoxia-induced EMT to promote metastasis in pancreatic cancer via miR-125a-3p to indirectly enhance the expression of RhoA [34]. HIF-1α is closely associated with the progression and metastasis of cancers, such as gastric cancer, hepatocellular carcinoma and pancreatic cancer [86–88]. Emerging evidence has revealed the molecular mechanism by which lncRNAs regulate HIF-1α expression. Some lncRNAs have been confirmed to be induced by HIF under hypoxic conditions, thus promoting tumor invasion, metastasis and EMT, such as lncRNA RP11-390F4.3 [89], MAPKAPK5-AS1 [90], PCED1B-AS1 [91], FEZF1-AS1 [92], ENST00000480739 [93], PCGEM1 and BX111 [94]. These hypoxia-induced lncRNAs could regulate EMT regulators to facilitate EMT in cancer. A new hypoxia-induced lncRNA, RP11-390F4.3, enhances EMT and metastasis by upregulating multiple EMT regulators, including Snail, Twist1, ZEB1 and ZEB2 [89]. LncRNA RP11-390F4.3 can also be activated directly by HIF-1α, which can bind to the three putative hypoxia response elements (HREs) located in the lncRNA RP11-390F4.3 proximal promoter [89]. HIF-1α could directly bind to the promoter of MAPKAPK5-AS1 to activate gene transcription, and ectopic expression of lncRNA MAPKAPK5-AS1 under hypoxia could promote hepatocellular carcinoma growth, metastasis and EMT [90]. Upregulated MAPKAPK5-AS1 enhances PLAG1-like zinc finger 2 (PLAGL2) expression by serving as a ceRNA to sponge miR-154-5p, subsequently activating EGFR/AKT signaling [90]. LncRNA PCED1B-AS1 promotes proliferation, invasion and EMT of pancreatic cancer cells by regulating the miR-411-3p/HIF-1α axis in pancreatic cancer by inducing the upregulation of HIF-1α [91]. LncRNA FEZF1-AS1 can act as an oncogene to enhance pancreatic cancer cell proliferation and invasion through the miR-142/HIF-1α axis under hypoxic conditions [92]. LncRNA ENST00000480739 may suppress pancreatic cancer invasion, metastasis and EMT by indirectly inhibiting HIF-1α expression [93]. The interaction between lncRNAs and HIF-1α may greatly impact tumor progression. A better understanding of the molecular mechanisms and biological functions of lncRNAs and HIF-1α will help find new effective anticancer strategies and novel tumor markers (Fig. 2).

## Therapeutic potential of lncRNA regulation in metastasis control as an appropriate therapeutic target for EMT in pancreatic cancer

A deeper understanding of lncRNAs will provide a unique opportunity to design better therapeutic strategies. Over the past decade, there has been substantial progress in the clinical application of RNA-based therapies, primarily including antisense oligonucleotides (ASOs) and small interfering RNAs (siRNAs), several of which have been approved by the FDA. Currently, many RNA therapies (such as miRNA mimics and anti-miRNAs) are undergoing clinical trials, but no lncRNA-based therapeutics have entered clinical trials [95]. LncRNA MALAT1 is differentially expressed in a variety of tumors, such as lung cancer, breast cancer, gastric cancer, prostate cancer and pancreatic cancer, and can promote tumor progression and play an important role in the regulation of cancer-related pathways [96]. In a xenograft lung cancer metastasis model, ASO blocking MALAT1 can markedly reduce metastasis [97]; thus, silencing MALAT1 may be an appropriate therapeutic target, and MALAT1 can be therapeutically targeted by ASO and siRNA. Specifically, in PDAC xenograft models, Tasaki et al. found that the targeting of TUG1 by ASO combined with their new cancer-specific drug delivery system could effectively reduce drug resistance and the systemic adverse effects of chemotherapy [98]. Therefore, it is expected that targeting these lncRNAs in combination with standard chemotherapy drugs may become a novel potent therapeutic option for patients with cancer. However, lncRNA-based therapies will face the following challenges, such as specificity of targeting, efficiency of delivery and tolerability of patients. Considering these challenges, a promising and innovative therapeutic approach will undoubtedly lead to unprecedented advances in the treatment of pancreatic cancer.

## Conclusions and perspectives

Although we have summarized in this review our understanding of lncRNAs as regulators of EMT in pancreatic cancer, the molecular mechanisms and contexts that define how tumors regulate each pathway to drive malignant behaviors in a lncRNA-dependent manner still remain poorly elucidated and require further study. The potential of EMT to stimulate the metastatic cascade during malignant progression has inspired researchers to explore EMT modulation as a cancer therapy strategy. Thus, a comprehensive understanding of lncRNA-mediated EMT, in which lncRNAs may exert tumor-promoting or tumor-suppressive roles, will improve our ability to appropriately target this pathway and seek pharmacologic therapy. These dysregulated lncRNAs are closely related to EMT, and it should be possible to identify novel and powerful biomarkers for tumor metastasis by testing the levels of core lncRNAs. Interestingly, exosome-encapsulated lncRNAs secreted from cancer cells function as messengers for cell-to-cell message communication and thereby control tumor progression by altering cellular metabolic pathways or remolding the tumor microenvironment [84, 99]. LncRNA HULC is considered an inducer of EMT and can be transferred by PDAC-derived EVs to modulate tumor metastasis and progression [41]. Therefore, finding EMT-related exosome-encapsulated lncRNAs and identifying their characteristics and effects on pancreatic cancer metastasis may provide new insights and potential treatment strategies for prevention, diagnosis and treatment.

## Data Availability

Not applicable.

## Abbreviations

ASO: Antisense oligonucleotides
APC: Adenomatous polyposis coli
AXIN2: Axis inhibition protein 2
CK1: Casein kinase 1
EMT: Epithelial-to-mesenchymal transition
EMT-TFs: EMT-activating transcription factors
GSK3: Glycogen synthase kinase-3
HGF: Hepatocyte growth factor
HIF-1α: Hypoxia-inducible factor-1α
LncRNAs: Long noncoding RNAs
MET: Mesenchymal-epithelial transition
miRNA: MicroRNA
m6A: N6-methyladenosine
PDAC: Pancreatic ductal adenocarcinoma
PDGF: Platelet-derived growth factor
siRNA: Small interfering RNA
Snail: Snail family transcriptional repressor
Twist: Twist family BHLH transcription factor
TGF-β: Transforming growth factor beta
TGF-βRI/TGF-βRII: TGF-β type I and II receptors
VEGF: Vascular endothelial growth factor
ZEB: Zinc-finger E-box-binding homeobox
